# Quantifying the impact of SpaceOAR hydrogel on inter‐fractional rectal and bladder dose during 0.35 T MR‐guided prostate adaptive radiotherapy

**DOI:** 10.1002/acm2.13344

**Published:** 2021-08-02

**Authors:** Reza Farjam, Sean S. Mahase, Shu Ling Chen, Madeline Coonce, Ryan T. Pennell, Ryan Fecteau, Bilal Chughtai, J. Keith Dewyngaert, Josephine Kang, Silvia Ch Formenti, Himanshu Nagar

**Affiliations:** ^1^ Department of Radiation Oncology Weill Cornell Medical College New York NY USA; ^2^ Department of Urology Weill Cornell Medical College New York NY USA

**Keywords:** 0.35 T MRI‐Linac, adaptive radiotherapy, SpaceOAR

## Abstract

**Purpose:**

To investigate the impact of rectal spacing on inter‐fractional rectal and bladder dose and the need for adaptive planning in prostate cancer patients undergoing SBRT with a 0.35 T MRI‐Linac.

**Materials and Methods:**

We evaluated and compared SBRT plans from prostate cancer patients with and without rectal spacer who underwent treatment on a 0.35 T MRI‐Linac. Each group consisted of 10 randomly selected patients that received prostate SBRT to a total dose of 36.25 Gy in five fractions. Dosimetric differences in planned and delivered rectal and bladder dose and the number of fractions violating OAR constraints were quantified. We also assessed whether adaptive planning was needed to meet constraints for each fraction.

**Results:**

On average, rectal spacing reduced the maximum dose delivered to the rectum by more than 8 Gy (*p* < 0.001). We also found that D_3cc_ received by the rectum could be 12 Gy higher in patients who did not have rectal spacer (*p* < 9E‐7). In addition, the results show that a rectal spacer can reduce the maximum dose and D_15cc_ to the bladder wall by more than 1 (*p* < 0.004) and 8 (*p* < 0.009) Gy, respectively. Our study also shows that using a rectal spacer could reduce the necessity for adaptive planning. The incidence of dose constraint violation was observed in almost 91% of the fractions in patients without the rectal spacer and 52% in patients with implanted spacer.

**Conclusion:**

Inter‐fractional changes in rectal and bladder dose were quantified in patients who underwent SBRT with/without rectal SpaceOAR hydrogel. Rectal spacer does not eliminate the need for adaptive planning but reduces its necessity.

## 1 INTRODUCTION

Prostate cancer is the most common, non‐cutaneous cancer among men with nearly 248 000 new cases and 34 000 deaths estimated to occur in 2021.^1^ Treatment options for localized prostate cancer include active surveillance, surgery, and radiation therapy.^2^ At a median of 10 years, prostate cancer‐specific mortality was low irrespective of the treatment assigned, with no significant difference among treatments.^3^ Radiation therapy for prostate cancer using intensity‐modulated and image‐guided techniques has decreased the frequency of treatment‐related adverse events.^4^, ^5^, ^6^ To date, moderate hypofractionation is the standard of care for localized prostate cancer,^7^, ^8^ but National Comprehensive Cancer Network (NCCN) Guidelines^9^ suggest that extreme hypofractionated treatment could be considered as a potential option in centers with appropriate technology and expertise. Hence, stereotactic body radiotherapy (SBRT) has been increasingly adopted for the treatment of intact prostate cancer with a 3‐fold increase observed in the United States between 2004 and 2012.^10^, ^11^

Stereotactic body radiotherapy provides tumor control comparable to those of conventional and hypofractionated radiotherapy.^12^, ^13^, ^14^ Men undergoing SBRT may experience new‐onset or worsening lower urinary tract symptoms (LUTS) and rectal toxicity^15^ depending on the extent of radiation. This implies that minimizing the radiation dose to nearby organs while keeping the same rate of tumor control is of great interest.

Multiple rectal displacement systems exist to minimize the dose to the rectum and alleviate the adverse events. The SpaceOAR system (Boston Scientific) is a Food and Drug Administration (FDA)‐approved, absorbable polyethylene glycol (PEG) hydrogel that is implanted before radiation therapy to reduce radiation exposure to the rectum. The injection of SpaceOAR is reported as an easy procedure^16^ and performed under transrectal ultrasound via the transperineal approach.^17^ Reported benefits include improved rectum, bowel, and genitourinary quality‐of‐life measures both in photon and proton therapy.^18^, ^19^ SpaceOAR has been shown to be very effective in lowering the incidence of grade 1 and 2 rectal toxicity as well as grade 1 urinary incontinence.^18^ Additionally, MRI linear accelerators (MRI‐Linac) are now available for real‐time imaging and on‐table adaptive radiotherapy that can take inter‐fractional anatomical changes into account leading to a better dosimetric outcome. Cuccia et al. have also shown the dosimetric benefit of SpaceOAR in the treatment planning of prostate cancer patients treated with a 1.5 T MRI‐Linac.^20^ In addition, the 0.35 T MRI‐Linac (ViewRay Inc.) is currently equipped with a robust tracking technology that enables us to use a tighter margin (2 mm compared to 5 mm without tracking technology) needed to account for setup error and minimize the radiation to nearby organs during the treatment which has shown promising results to lower the genitourinary (GU) and gastrointestinal (GI) toxicity.^21^ Hence, to further establish our protocol for MR‐guided adaptive radiotherapy (MRgART) of prostate cancer patients, we aimed to extend our understanding of the role of SpaceOAR in facilitating the on‐table adaptive radiotherapy process. In MRgART using a 0.35 T MRI‐Linac, after patient positioning, a new MRI scan is acquired and used for target delineation and organs at risk (OARs) contouring. The plan from the simulated image is then loaded and re‐calculated for the new MRI scan and dose to the new contours are updated. If the new dose distribution does not meet the dosimetric constraints, the plan will be adapted until all conditions are satisfactorily met. The treatment starts after the physics team performs all the quality assurance checks including the dose distribution and plan quality, independent monitor unit calculation, and also verifying the newly contoured structures. Right before the treatment, a tracking target (in our case, prostate gland plus a portion of proximal seminal vesicle) is contoured by the user which will be used for gating. During the treatment and using real‐time imaging, the tracking target is automatically segmented by the system, and radiation is delivered whenever the segmented target is at the baseline position. Adaptive process is a complex and very lengthy process and may take more than 90 min depending on the situation and could also be very cumbersome for the patient. Considering that 67% of our total patients treated with MRI‐Linac are a prostate cancer patient, identifying those who greatly benefit from adaptive therapy is very crucial and could save time from our radiation oncology team, the non‐adaptive case may take only 30–45 min, increase the machine throughput, and also improve the patient comfort. Therefore, we plan to investigate the role of SpaceOAR in MRgART in more detail in the current study. Since our major concern in radiotherapy of prostate cancer patients is the quality of life and tissue toxicity, we sought to study differences in inter‐fractional rectal and bladder dose and the necessity for adaptive planning in a retrospective analysis of 20 patients undergoing SBRT with a 0.35 T MRI‐Linac, 10 with and 10 without SpaceOAR.

## 2 MATERIALS AND METHODS

### 2.1 Patients

Two groups of 10 patients diagnosed with prostate cancer and underwent stereotactic body radiotherapy were included in this IRB‐approved retrospective study. All patients were treated with a dose of 36.25 Gy in five fractions to the prostate and seminal vesicles using a 0.35 T MR‐guided Radiotherapy (MRgRT) system. Nodal regions were not treated in these patients. In the first group (spacer group, aged 67–81 with a median of 75), transperineal rectal spacer (SpaceOAR) had been implanted prior to treatment. Rectal spacer was not utilized in the second group (non‐spacer group, aged 63–80 years with a median of 65.5). Rectal spacer is offered to all patients and its use was based on patient choice.

In our MRgRT workflow, each patient is initially simulated with both CT and MRI‐Linac. MRI simulator images are used for contouring and CT scans are used for dose calculation. TG101^22^ dosimetric constraints were used for the treatment planning of both groups. However, in cases where dosimetrists had substantial difficulty to meet TG101 dose constraints, they could use NRG‐GU005^23^ dose limits for treatment planning after consulting with a radiation oncologist. Also, a coverage of 98% for the prescription isodose line was initially sought for the planning target volume (PTV) but dosimetrists were allowed to plan with 95% coverage if they had difficulty to meet the organs at risk constraints. Target included the prostate gland as well as a portion of the proximal seminal vesicle. PTV was constructed by adding a 2 mm margin isotopically around the target. In the spacer group, 11 to 16 beam angles (mean: 13.4 ± 1.9) with 35 to 66 segments (mean: 56.1 ± 9.3) were used for planning. We used 9–16 beam angles (mean: 12.3 ± 1.9) with 41–66 segments (mean: 55.6 ± 8.9) for treatment planning in the non‐spacer group. No significant difference existed between the two groups in terms of the number of beam angles (*p* < 0.21) and segments (*p* < 0.9) used for treatment planning. They were the dosimetrists’ choice during planning.

### 2.2 On‐table data acquisition

Before a treatment starts, a new MRI scan is acquired and used for patient setup. The new scan is also used for target tracking during the treatment. If the setup is stable, this MRI scan is utilized for the entire treatment and radiation delivery continues with no interruption. In some cases where the patient moves substantially and the initial setup is distorted, multiple MRI scans may be acquired, and the treatment may resume after adjustment. If an interruption occurs, the system splits the plan into multiple MRI images. For simplicity and to include all plan components in one image, we only included uninterrupted fractions in our analysis. Therefore, to maximize the number of fractions in our study, we selected only patients who had at least four uninterrupted fractions in their treatment. From the total of 50 possible fractions, we found 46 uninterrupted fractions for each group of patients used in this study.

### 2.3 Organs at risk contouring

Structures surrounding the prostate gland are the rectum, bladder, bladder wall, large and small bowel, penile bulb, urethra, femoral heads, and skin. These structures were contoured initially for treatment planning. Since most of these structures are distant from the prostate and receive low dose, their inter‐fraction displacement has negligible toxicity consequences. We also consider a 3 mm margin around the urethra as planning at risk volume (PRV) to provide confidence regarding the dose fluctuation in the urethra. As the bladder and rectum are two nearby structures whose subtle disposition could lead to a substantial change in toxicity outcome, we focused our attention on the dosimetry of these two structures. Rectum and bladder were both manually contoured by radiation oncology residents (S.S.M. and R.F.) and then confirmed by a faculty radiation oncologist (H.N.) on each newly derived MRI image. Assuming 4mm thickness,^24^ the bladder wall was automatically contoured. The contouring procedure was performed for all 46 MRI images in each group.

### 2.4 Statistical analysis

As discussed earlier, TG101 and NRG‐GU005 dosimetric constraints were used during treatment planning. Table 1 briefly shows these constraints for rectum, bladder, and bladder wall, respectively.

**TABLE 1:** TG101 and NRG‐GU005 dosimetric constraint for rectum and bladder

Rectum (Gy)		Bladder Wall (Gy)	
D_0.03cc_	D_20cc_	D_0.03cc_	D_15cc_
TG101			
38	25	38	18.3

Rectum (Gy)					Bladder (Gy)	
D_0.03cc_	D_3cc_	D_10%_	D_20%_	D_50%_	D_0.03cc_	D_10%_
NRG‐GU005						
38–40	34.4–36	32.6–34	29–30	18.1–19	38–40	18.1–20

Numbers denote the acceptable range

To study the dosimetric impact of the transperineal rectal spacer in rectal and bladder dose, differences in the above dose constraints were initially evaluated in the original plans for both groups. Recalculating a plan on the newly acquired MRI scan for each fraction, the dose to the updated rectum, bladder, and bladder wall structures was then measured in the delivered plan. This procedure was performed for all 46 fractions in both groups. The cumulative dose to each structure was then calculated by adding the delivered dose from all fractions. Group‐wise dosimetric differences in the delivered plans were then investigated for each structure. Unpaired statistical Student *t*‐test was performed to find whether significant differences exist between the two groups. To assess inter‐fraction changes in the rectal and bladder dose, differences in the above dosimetric constraints were also calculated in each fraction and the necessity for adaptive planning was evaluated subsequently. As our main concern in treating prostate cancer patients is to lower the toxicity, we require a plan to be adapted if any of the bladder and rectum dosimetric constraints were not met in a fraction. For the sake of this analysis, we assume a plan is to be adapted if at least one of the bladder or rectum constraints mentioned in Table 1 was not met. We ultimately assessed the incidence of acute GU and GI toxicity in both groups of patients. Per RTOG, acute toxicity is defined as any radiation‐related toxicity that occurred within 90 days of the treatment start date.^25^ All patients were evaluated during, at the end of treatment, and 3 months after the radiation to evaluate their radiation‐induced acute toxicity. During each follow‐up session, the incidence of urinary and rectal complications including frequency, dysuria, hematuria, infections, and incontinence was assessed. Not all long‐term follow‐up data were available to assess the late effect toxicity.

## 3 RESULTS

Table 2 shows that the number of initial and delivered plans failed to meet dosimetric constraints in each patient cohort. For this analysis, we used each dose constraint as a hard threshold. As shown, NRG rectal dose constraints were met in initial plans for all patients but one in the non‐spacer group. Four patients failed to meet TG101 rectal dose limits in the non‐spacer group in contrast to one in spacer patients. In the delivered plans, rectum dose constraints were met in all spacer patients, but they exceeded the threshold in two patients who did not have a spacer, based on NRG‐GU005 criteria. Failure in delivered rectal dose in non‐spacer patients reached six cases based upon TG101 dose limits. As shown, more patients failed to meet bladder constraints in both groups, but failure was more prevalent in patients without a spacer.

**TABLE 2:** Number of initial and delivered plans failed to meet dosimetric constraints for each patient cohort

	Rectum									Bladder NRG			BladderWall TG 101				
Spacer	D_0.03cc_ TG101	D_0.03cc_ NRG	D_3cc_	D_20cc_	D_10%_	D_20%_	D_50%_	Combined		D_0.03cc_	D_10%_	D_0.03cc_		D_15cc_	Combined		
								NRG	TG101						NRG		TG101
Initial Plans																	
Yes	1	0	0	0	0	0	0	0	1	0	2	2		3	2		5
No	4	0	1	0	0	0	0	1	4	0	6	8		10	6		10
Delivered Plans																	
Yes	0	0	0	0	0	0	0	0	0	0	4	5		2	4		6
No	6	0	1	0	0	0	1	2	6	2	9	9		8	9		9

The combined column represents the number of patients who failed to meet either TG101 or NRG dose constraints. Failure happens if at least one metric passes the threshold for a structure.

**FIGURE 1:**
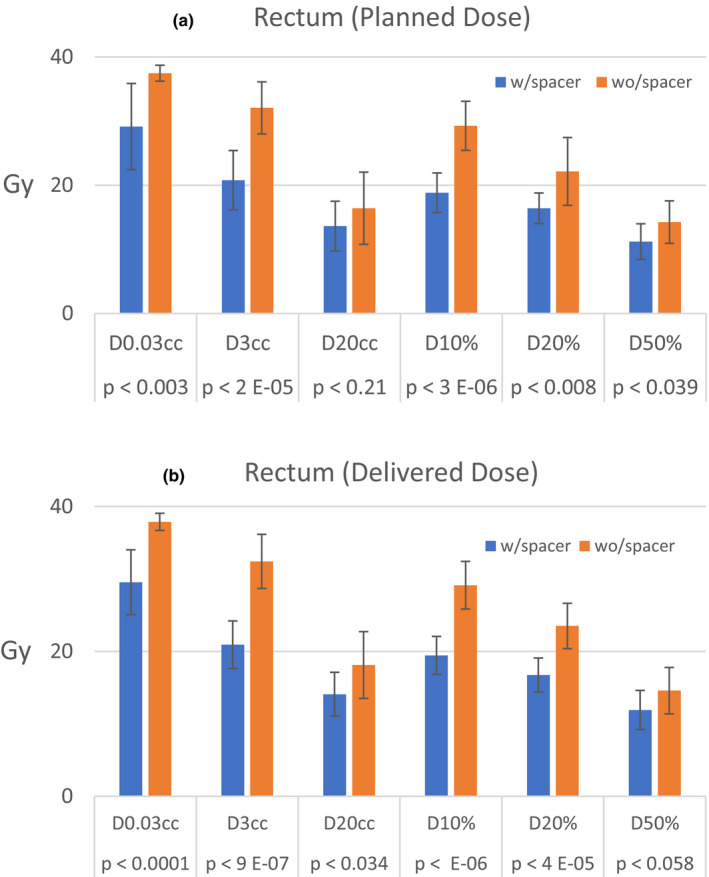
Comparison results of the rectal dose constraints in initial and delivered plans in patients with and without the rectal spacer

**FIGURE 2:**
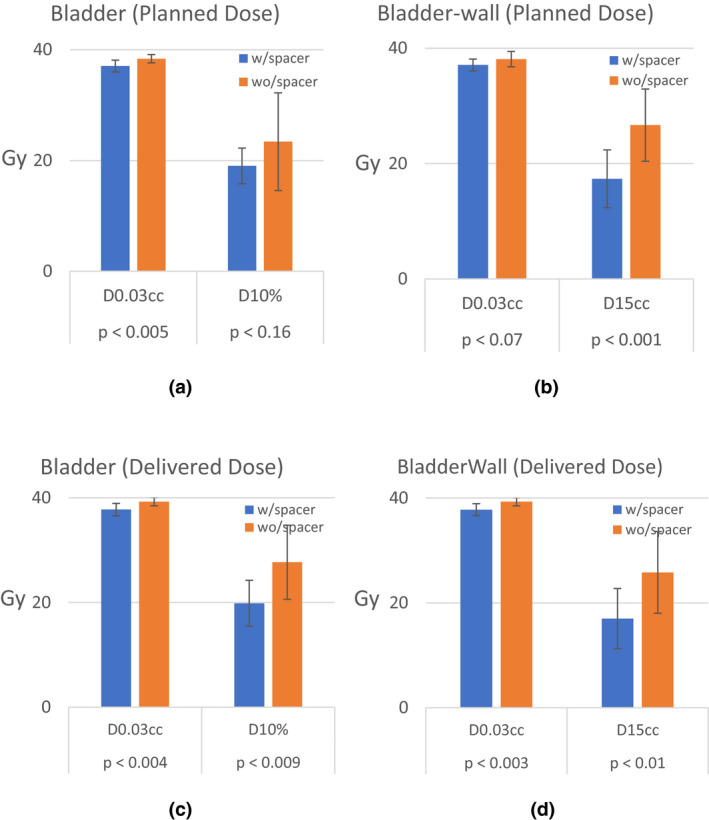
Comparison results of the bladder and bladder wall dose constraints in initial and delivered plans in patients with and without the transperineal rectal spacer

Figures 1, 2 and 1, 2 illustrate a comparison result of the planned and delivered dose to the rectum, bladder, and bladder wall structures for two groups of patients, respectively. As shown, the maximum and volumetric dose to both rectum and bladder were significantly lower in patients with rectal spacer. Figure 1a shows that the maximum planned dose to the rectum could be more than 8 Gy higher in patients who did not have spacer compared to those with spacer (*p* < 0.003). When it comes to D_3cc_, the difference could be more than 12 Gy (*p* < 2E‐05). Figure 2 shows a similar trend for the delivered dose, that is, it shows that rectal spacing reduced the maximum dose delivered to the rectum by more than 8 Gy (*p* < 0.001). It also shows that D_3cc_ received by the rectum was 12 Gy higher in non‐spacer patients (*p* < 9E‐7).

**FIGURE 3:**
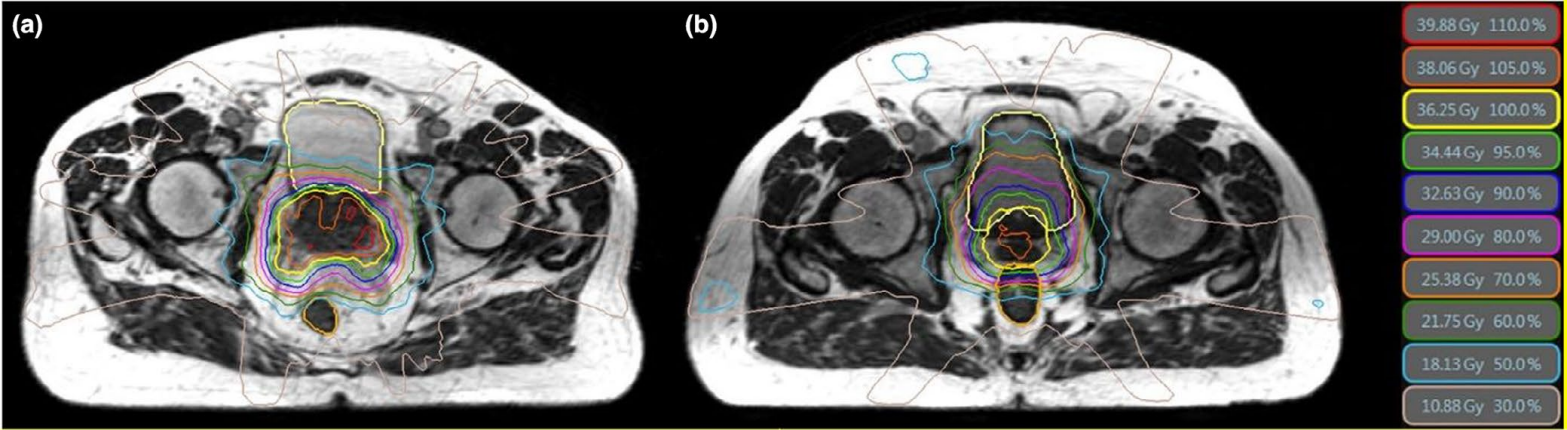
Examples of dose distribution on a spacer (a) and non‐spacer (b) patient. The bladder and rectum are contoured as yellow and orange structures in these two images

Figure 2a,b show that rectal spacer could also help to reduce the dose to the bladder and bladder wall although the difference was not as high as what we observed in the rectum. Figure 2c,d show a comparison result of the final dose delivered to the bladder and bladder wall for the two groups of patients, respectively. The maximum delivered dose to bladder and bladder wall could be more than 1 Gy higher in patients without a spacer. They also show that D_15cc_ in the bladder wall could be more than 8 Gy higher in patients without the spacer (*p* < 0.01). Figure 3a,b also show examples of dose distribution on a spacer and non‐spacer patient and illustrate how favorable dosimetry could be achieved for spacer patients. The bladder and rectum are contoured as yellow and orange structures in these two images.

Table 3 shows the number of delivered fractions that failed to meet dosimetric constraints for each group of patients. As shown, rectum constraints were met in almost all fractions except one in patients with rectal spacer. In contrast, the incidence of dose constraint violation was higher for each metric in patients without the rectal spacer. This table also confirms that failure to meet bladder constraints occurred in both groups but was more prevalent in patients without the rectal spacer.

**TABLE 3:** Number of fractions failed to meet dosimetric constraints in each patient cohort

Spacer	Rectum							Bladder NRG		Bladder wall TG 101	
	D_0.03cc_ TG1011	D_0.03cc_ NRG	D_3cc_	D_20cc_	D_10%_	D_20%_	D_50%_	D_0.03cc_	D_10%_	D_0.03cc_	D_15cc_
Y	1	1	0	0	0	0	0	3	23	21	18
N	29	0	8	4	5	1	3	7	40	42	38

There were 46 fractions in each group

**FIGURE 4:**
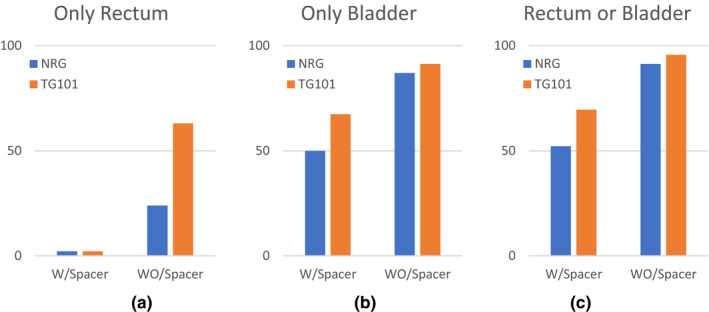
Percentage of fractions that failed to meet either rectum or bladder dose constraints. Vertical axis denotes percentage

Figure 4a‐c show the percentage of fractions in which re‐planning was needed due to the violation of bladder or rectum dose constraint for each set of dose constraints. Figure 4a shows that spacer‐patients met both TG101 and NRG dose constraints in all fractions except one (~2%). In contrast, it shows that in ~23% (11/46) of the fractions, at least one of the NRG rectal dose constraints exceeded the threshold in non‐spacer patients. The rate of failure in rectal dose was ~63% for TG101 dose constraints. Similarly, Figure 4b shows that, based on NRG criteria, the bladder dose constraints exceeded the threshold in 50% (23/46) of the fractions in patients with a spacer in contrast to ~86% (40/46) failure in the non‐spacer group. These values were ~67% (31/46) and ~91% (42/46), respectively, for TG101 dose constraints. Figure 4c also reveals that for patients in whom rectal spacer was implanted, only 52% = 24/46 of fractions needed to be re‐planned while this value was 91% = 42/46 for patients without spacer based upon NRG criteria. These numbers were ~69% (31/46) and ~95% (44/46) for TG101 dose limits. As adaptive planning requires new contours, re‐optimization, and QA, reducing the need for re‐planning could save time and lower the cost. Furthermore, as the whole adaptive process is performed while the patient is on the table, this leads to more patient comfort.

Finally, Table 4 shows the incidence of acute radiation‐induced GU and GI toxicities. As shown, an increase in the frequency of urination is the most prevalent form of acute toxicity and appeared mainly in the non‐spacer group. As also shown, 7 out of 10 patients with implanted spacer reported no urinary symptoms compared to only 1 patient in the non‐spacer group.

**TABLE 4:** Incidence of acute radiation‐induced toxicity in groups of the spacer and non‐spacer patients

Specific Toxicity		Spacer	Non‐spacer
GU	UTI (Urinary Tract Infection)	0	0
	Frequency	2	7
	Dysuria	0	2
	Hematuria	0	0
	Incontinence	0	0
	Hesitancy	0	1
	Nocturia	2	3
GI	Blood in stool	0	1
	Frequency/difficulty in defecation	2	3
# of Patients without GU toxicity		7	1
# of Patients without GI toxicity		8	6

GU, genitourinary; GI, gastrointestinal.

## 4 DISCUSSION

Stereotactic body radiotherapy has been increasingly used in the treatment of prostate cancer and shown to provide tumor control Comparable to conventional and hypofractionated radiotherapy.^12^, ^13^, ^14^ Radiation‐induced gastrointestinal (GI) and genitourinary (GU) toxicities are the main cause of concern in men undergoing SBRT. In recent years, 0.35 T MRI‐Linac (ViewRay Inc.) has provided unprecedented tools for adaptive planning and target motion tracking which has been shown^21^ to be very effective in reducing the GU and GI toxicity. Using MRI‐Linac, inter‐and intra‐fraction changes in structures’ position and deformation are accounted for enabling us to adjust the dosimetry favorably. This lowers the chance of constraint violation and target mis‐irradiation leading to a better outcome and lower radiation‐induced toxicity. In a recent study, Tetar et al. and colleagues presented^26^ patient‐ and clinician‐reported outcomes from a prospective clinical trial at 1 year following stereotactic MR‐guided radiation therapy in patients with localized prostate cancer and without a rectal spacer. Based upon the patient questionnaires and recorded adverse effects, the most significant urinary and bowel symptoms were seen in the first 6 weeks of follow‐up. All symptoms decreased and returned to baseline values at 12 months of follow‐up. No grade ≥3 toxicity was reported for these cases. The authors reported an initial increase in the QLQ‐PR25^27^ symptom scores (five conditional questions assessing urinary and bowel symptoms, sexual activity, and functioning as well as urinary incontinence) both at the end of MRgRT and at the 6‐weeks follow‐up. Although these results are promising, the authors noted that longer follow‐up is needed. Multiple rectal displacement systems also exist to minimize the dose to the rectum and alleviate the treatment adverse events. Zelefksy et al. have shown^28^ that patients with a rectal spacer placement experienced significantly less late rectal toxicity (1% versus 6%). We presumed that the concurrent use of MRgRT and transperineal rectal spacer could provide more favorable dosimetry and facilitate the adaptive radiotherapy workflow. Hence, we studied differences in rectal and bladder dose and a necessity for adaptive planning in patients undergoing SBRT with a 0.35 T MRI‐Linac with and without rectal spacing.

Our study shows that implanting a rectal spacer plays a substantial role in lowering both maximum and volumetric dose to both rectum and bladder significantly. We found that the maximum dose to the rectum could be more than 8 Gy higher in patients without a spacer. For D_3cc_, this difference could be more than 12 Gy. Similar trends were also observed for D_20cc_, D_10%_, D_20%_, and D_50%_, respectively. Interestingly, we observed that the maximum dose to bladder and bladder wall could also be significantly higher in patients who did not have rectal spacer. We also noticed that D_15cc_ in the bladder wall could be more than 8 Gy higher in patients without the spacer. This reveals that rectal spacer is not only effective to spare the rectum, but also helps to reduce the dose to the bladder. Our investigation shows that when the spacer is implanted, the isodose lines are pushed toward the rectum lowering the dose to the bladder and bladder wall as well which has been clearly shown in Figure 3. This may also lower the chance of inter‐fraction bladder constraint violation in patients who had spacer.

In addition to favorable dosimetry, we also realized that implanting the rectal spacer could facilitate the process of on‐table adaptive planning and reduce the necessity to adapt in patients with rectal spacer. Since one of the lengthiest parts of adaptive radiotherapy is re‐optimization, this may reduce the total time needed for a patient to lie on the table. In our institution, treating a non‐spacer case can take between 30 and 45 min. Adaptive planning can prolong this process substantially and could double the time. Also, one major point that needs to be considered for adaptive planning is that the dose to each organ could be measured in each fraction and compared with the baseline plan. Currently, we consider each fraction individually but considering the dose from the previously delivered fraction may also help to calculate the allowed dose for the remaining fractions and skip adaptive planning if not really needed. In addition, as implanting a rectal spacer could also cost less than billing for five adaptive plans, it could be also more cost‐effective for the patients at a trade‐off of small procedural risk.

As stated earlier, one major advantage of 0.35 T MRI‐Linac is its unique image‐guidance ability to track the target and gate the treatment. This new feature has almost eliminated the concern of target motion during the treatment as the dose delivery only happens while the target is at the baseline position.^29^ However, we envisioned that SpaceOAR could have an impact on the total treatment time by affecting the motion of the target. Nonetheless, we observed no significant changes in the treatment time between the spacer and non‐spacer groups due to differences in the gating time. For this purpose, for each group of patients, we subtracted the total beam‐on time (provided by the system) from the total time (provided by the delivery cine) and measured the differences. On average, gating prolonged the treatment time for about ~170 and ~177 s for the spacer and non‐spacer groups, respectively, with no significant difference between the two cohorts (*p* < 0.8). This assessment is in accordance with the initial finding that differences in intrafraction motion in patients with and without spacer were both within measurement uncertainty (<1 mm) and the addition of a rectal spacer does not eliminate the need for intrafraction motion management.^30^

In the current study, we focused our attention on the dosimetric differences in rectum and bladder structures when the rectal spacer was implanted. However, our initial assessment has shown that the incidence of acute toxicity is also more prevalent in non‐spacer patients. It is worthwhile to mention that at the time of this study, not enough long‐term follow‐up data were available for late toxicity assessment between the two groups. Hence, in our future work, we plan to assess both acute and late toxicity effects in a larger dataset.

## 5 CONCLUSION

In this work, we investigated differences in rectal and bladder dose and necessity for adaptive planning in patients undergoing SBRT on an MRI‐Linac with and without rectal spacing. We found that rectal spacer lowers the maximum and volumetric dose in both rectum and bladder, significantly. However, it does not eliminate the need for adaptive planning in the case of ablative radiotherapy but reduces the necessity to adapt significantly. Also, as adaptive planning requires new contours, re‐optimization, and QA, reducing the need for re‐planning could save time from the radiation oncology crew, lower the treatment cost, and improve patient comfort.

## 6 DATA AVAILABILITY STATEMENT

Authors elect to not share data.

## CONFLICT OF INTEREST

None of the authors have any conflict related to this work.
